# Association Between Entropy Monitoring, Burst Suppression and Early Postoperative Cognitive Dysfunction in Emergency Surgery: A Retrospective Cohort Study

**DOI:** 10.3390/jcm15030968

**Published:** 2026-01-25

**Authors:** Liliana Mirea, Ana Maria Dumitriu, Cristian Cobilinschi, Bogdan Cristian Dumitriu, Raluca Ungureanu, Cosmin Andrei Andrei, Răzvan Ene, Dragoș Ene, Radu Țincu, Ioana Marina Grințescu

**Affiliations:** 1 Faculty of Medicine, “Carol-Davila” University of Medicine and Pharmacy, 050474 Bucharest, Romania; llmirea@yahoo.com (L.M.); ioana.grintescu@rospen.ro (I.M.G.); 2 Anesthesiology and Intensive Care Clinic, Clinical Emergency Hospital Bucharest, 041915 Bucharest, Romania; 3 Department of Surgery, Clinical Emergency Hospital Bucharest, 041915 Bucharest, Romania; 4 Orthopedics and Trauma Surgery, Clinical Emergency Hospital Bucharest, 041915 Bucharest, Romania; 5 Clinical Toxicology, Clinical Emergency Hospital Bucharest, 041915 Bucharest, Romania

**Keywords:** entropy-guided anesthesia, burst suppression, emergency surgery, postoperative delirium, NEECHAM confusion scale

## Abstract

**Background/Objectives:** Emergency surgical patients are at increased risk of acute postoperative delirium. Processed EEG monitoring, such as entropy indices and burst suppression ratio (BSR), may optimize anesthetic dosing, yet their role in non-elective surgery remains underexplored. This retrospective cohort study aimed to examine whether entropy monitoring and intraoperative burst suppression are associated with the incidence of early postoperative delirium during the first 72 h after emergency surgery. **Methods:** Adult patients undergoing emergency surgery between March 2022 and March 2024 were classified into two groups based on anesthesia records: the entropy-monitored group (EG) and the standard care group without processed EEG (SG). Demographic, intraoperative, and cognitive data (NEECHAM scores during the first 72 h) were extracted from institutional perioperative records. The primary outcome was postoperative delirium (NEECHAM ≤ 24), with secondary analyses examining anesthetic exposure, burst suppression, and intraoperative hemodynamics. **Results:** Entropy-monitored patients received lower sevoflurane and fentanyl doses and exhibited improved hemodynamic stability, including fewer hypotensive episodes and lower norepinephrine requirements. Early postoperative cognitive dysfunction (NEECHAM ≤ 24) was more frequent among patients with intraoperative burst suppression, with BSR > 15% or suppression duration > 6 min strongly associated with cognitive decline within the first 72 h. **Conclusions:** In this retrospective cohort, entropy-guided anesthesia was associated with more precise anesthetic titration and more stable hemodynamic parameters. Burst suppression characteristics may serve as indicators of neurocognitive vulnerability rather than solely reflecting direct effects of anesthetic dosing. These results support the use of processed EEG monitoring in emergency surgery, though prospective studies are needed to confirm these findings.

## 1. Introduction

Perioperative optimization in surgical patients aims not only to maintain intraoperative physiological stability, but also to support postoperative recovery and improve both functional and clinical outcomes. However, most published perioperative management recommendations predominantly focus on elective surgical interventions involving carefully prepared and pre-optimized patients [1,2,3]. The specialized literature abounds with recommendations for stratifying and managing cardiovascular events, although there are other, more frequent postoperative complications that contribute to prolonged hospital stays and, implicitly, increased morbidity. Among these, perioperative cognitive dysfunctions constitute a significant proportion of immediate or delayed postoperative complications in surgical populations, with an overwhelming prevalence among the elderly [4,5].

Based on findings from studies conducted in elective surgery cohorts, the most recent guidelines from the European Society of Anaesthesiology and Intensive Care (ESAIC) recommend the use of EEG-based neuromonitoring to guide anesthetic depth and reduce the risk of postoperative delirium [2]. Furthermore, index-based EEG monitoring including the Burst Suppression Ratio (BSR) may enhance anesthetic titration. BSR quantifies alternating periods of cortical activity and inactivity, hallmarks of deep anesthetic states [6]. Ideally, the percentage of BSR during general anesthesia should be minimal, but the incidence increases if the anesthesia is too deep. Prolonged or frequent burst suppression has been associated with increased likelihood of postoperative delirium in multiple studies [7,8], although thresholds for clinically relevant BSR exposure remain uncertain, and susceptibility to suppression may itself reflect underlying frailty or neurocognitive vulnerability [9].

Emergency surgery presents a fundamentally different clinical setting. Time for preoperative optimization is limited, baseline physiological stress is higher, and patient vulnerability is amplified. The role, practicality, and potential benefit of processed EEG-based depth monitoring in urgent, non-elective procedures remain insufficiently investigated. While the theoretical basis for neuromonitoring is strong, its actual perioperative impact in emergency settings is not well established.

To address this knowledge gap, we conducted a retrospective cohort analysis of adult emergency surgery patients, comparing cases in which entropy monitoring was employed versus procedures performed without EEG-guided depth assessment. We evaluated anesthetic dosing patterns, hemodynamic stability, incidence of burst suppression, and early postoperative cognitive outcomes. We hypothesized that entropy-monitored cases would demonstrate different anesthetic titration profiles and that burst suppression metrics would correlate with early postoperative cognitive dysfunction.

## 2. Materials and Methods

### 2.1. Study Design and Ethical Approval

This retrospective cohort study was conducted at the Clinical Emergency Hospital Bucharest and adhered to institutional ethical and data-protection standards. The analysis included perioperative records from March 2022 to March 2024. Institutional approval for retrospective analysis of anonymized clinical data was obtained from the Research Ethics Committee of the Clinical Emergency Hospital Bucharest (approval ID 10308/2025). The study complied with GDPR-aligned institutional policy, and individual patient consent was waived, as all analyzed data were originally collected as part of routine clinical care and were fully anonymized prior to analysis.

### 2.2. Patient Selection

Adult patients (≥18 years, ASA I–IV) who underwent major abdominal or orthopedic emergency surgery under general anesthesia during the study period were eligible for inclusion. Cases were identified through surgical schedules, OR procedure logs, and archived anesthetic documentation. Inclusion was based exclusively on available documentation, and no additional patient contact or follow-up outside routine care occurred.

Patients were excluded if any of the following were noted in operative or postoperative records:

- severe craniocerebral trauma,
- documented untreated pre-existing cognitive dysfunction or active psychiatric pathology,
- intraoperative ketamine administration,
- surgical duration < 60 min,
- reintervention within 72 h,
- major perioperative neurological, cardiovascular, or metabolic complications,
- inability to undergo extubation in the immediate postoperative period, precluding cognitive assessment.

Patients were subsequently categorized based on the intraoperative use of processed EEG:

1. Entropy-monitored cohort—cases in which entropy-based monitoring was applied during anesthesia,
2. Standard-care cohort—cases managed without processed EEG.

### 2.3. Entropy Monitoring

In cases where processed EEG monitoring was used in routine practice, entropy values were obtained using the GE Entropy™ Module (GE Healthcare Finland Oy, Helsinki, Finland) with standard forehead sensors. These sensors consist of adhesive electrodes placed on the forehead, above the eyebrows and lateral to the midline, ensuring optimal detection of frontal EEG and electromyographic signals. Monitoring was initiated after securing venous access and continued throughout the intraoperative period. The monitor displayed both state entropy (SE), a more stable parameter, and response entropy (RE), which reacts faster [10].

SE values range from 0 to 91 and primarily reflect cortical EEG activity correlating with hypnotic depth, being unaffected by frontal muscle activity or neuromuscular blockers [11,12]. RE values range from 0 to 100 and incorporate faster electromyogram (EMG) components, being sensitive to facial muscle activation with a reaction time under two seconds [12]. RE during general anesthesia can occur after painful stimuli and may indicate insufficient analgesia, as neuromuscular blockers do not fully suppress frontalis EMG activity [13,14].

The entropy module also displays BSR, indicating periods of suppressed neuronal activity as a percentage of total monitoring time [10]. When burst suppression occurs, spectral and response entropy values are calculated as if representing a superficial hypnosis level [15]. BSR is ideally minimal during anesthesia but increases with excessive depth, typically when entropy falls below 40 [13]. BSR estimation requires at least one minute of recording to avoid fluctuations [13].

For this analysis, the following entropy-derived data were extracted from anesthesia records:

- recorded SE and RE values,
- documentation of BSR events ≥ 1 min in duration,
- total BSR exposure expressed as percentage of monitoring time.

Use of entropy was not standardized or protocol-driven; rather, its application reflected individual clinical judgment and availability of monitoring equipment.

Accordingly, entropy-derived variables reflect real-world heterogeneity in monitoring practice rather than a uniform decision algorithm.

### 2.4. Anesthesia

All patients received general anesthesia delivered according to standard institutional emergency-surgery protocols. Induction generally consisted of fentanyl, propofol titrated to clinical effect, succinylcholine for rapid sequence intubation, and rocuronium for neuromuscular relaxation. Maintenance typically consisted of sevoflurane in an air–oxygen mixture, with ventilation adjusted to maintain ETCO_2_ between 35 and 45 mmHg.

In cases without entropy monitoring, titration of anesthetic depth followed routine clinical parameters such as MAC and hemodynamic response. In cases where entropy was used, anesthesiologists could incorporate SE and RE values to guide anesthetic adjustment; however, dosing decisions ultimately reflected real-world clinical judgment.

Because the study was retrospective, no standardized anesthetic protocol was mandated, and intraoperative drug administration patterns were inferred from charted dosing records.

Instances where dosing information was missing, ambiguous, or incompletely charted were handled using case-exclusion for that specific variable rather than data imputation.

### 2.5. Data Collection

Data extraction was performed from routinely documented perioperative records, including anesthetic forms, vital-sign logs, surgical reports, medication logs, and postoperative nursing assessments. Variables collected included demographics (age, sex, BMI), comorbidities, preoperative laboratory values, intraoperative anesthetic dosing, fluid and transfusion volumes, and vasopressor use.

Burst suppression data (presence, duration, and BSR%) were extracted from available entropy recordings when present. Hemodynamic events were interpreted based on documented vital sign trends, using the following operational definitions:

- Mean arterial pressure < 65 mmHg for ≥2 consecutive readings
- SBP > 140–160 or DBP > 90–95 mmHg
- Bradycardia < 60 bpm
- Tachycardia > 90 bpm

Postoperative pain scores using VAS were extracted when available.

### 2.6. Delirium Assessment

Postoperative cognitive status during the first 72 h was assessed using the NEECHAM Confusion Scale, as documented in the clinical records by ICU nursing and physician staff involved in routine patient care. The NEECHAM scale was selected for its comprehensive evaluation of cognitive processing (attention, orientation, information processing), behavioral function (motor activity, verbal communication, appearance), and physiological control (vital signs, oxygen saturation, urinary continence) [16,17,18,19]. Scores range from 0 to 30, with lower scores indicating greater cognitive impairment.

The NEECHAM Confusion Scale was chosen because it enables early detection of both hyperactive and hypoactive delirium subtypes, which are common in emergency surgical populations. While other tools such as the CAM-ICU and ICDSC are widely used, the NEECHAM scale’s integration into routine nursing assessments at our institution facilitated systematic and practical delirium monitoring in this high-acuity setting.

For this study, early postoperative cognitive dysfunction was defined as a NEECHAM score ≤ 24, based on established categories:

- 0–19: delirium
- 20–24: confusion syndrome
- 25–26: risk for confusion
- 27–30: normal cognition

Since assessments were conducted as part of routine clinical care in a real-world emergency surgery setting rather than under controlled research conditions, evaluations were generally performed once daily. Any variability in the timing of these assessments was accounted for in the statistical analysis.

### 2.7. Statistical Data Analysis

Statistical analysis was performed using SPSS version 26. Descriptive statistics were used to summarize the dataset, including means and standard deviations for normally distributed variables, and medians with interquartile ranges for skewed data. Group comparisons between entropy-monitored and standard-care patients were performed using independent-samples *t*-tests or Mann–Whitney U tests for continuous variables, and chi-square or Fisher’s exact tests for categorical variables, as appropriate.

Associations between entropy use and anesthetic dosing, vasopressor administration, fluid volume, and hemodynamic parameters were explored using univariate and multivariable regression models. For binary outcomes (e.g., vasopressor use), logistic regression models were applied. For ordinal outcomes (e.g., number of hypotensive episodes), ordinal logistic regression was used. Effect estimates were expressed as mean differences, odds ratios, or regression coefficients with 95% confidence intervals.

Postoperative NEECHAM scores at 24, 48, and 72 h were compared between groups descriptively and using within-subject repeated-measure comparisons where documentation permitted. Correlations between burst suppression metrics and cognitive outcomes were assessed using Pearson or Spearman correlation coefficients.

As this was a retrospective analysis, missing data were handled using listwise exclusion for the specific variable analyzed, with no imputation of unavailable values. Statistical tests were two-sided, with *p* < 0.05 considered significant for exploratory interpretation.

## 3. Results

### 3.1. Patient Characteristics and Intraoperative Anesthetic Management

After applying exclusion criteria, 168 patients met inclusion criteria. Baseline clinical characteristics were comparable between the entropy-monitored (EG) and standard-care (SG) cohorts, with no differences in age distribution, ASA class, or comorbidity burden (Table 1).

Entropy-guided patients received consistently lower doses of volatile anesthetic and intraoperative fentanyl, as shown in Table 2. Differences in rocuronium use were modest and dependent upon surgical category. Fluid administration and blood product use were similar between groups, though slight reductions in crystalloid and vasopressor requirements were observed in the EG group (Table 2 and Table 3).

**Scheme 1:**
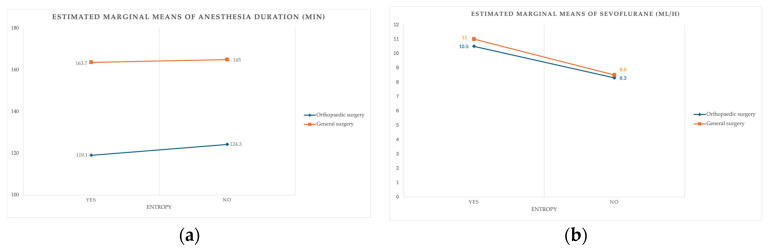
(**a**) Relationship between procedure type, monitoring status, and anesthesia duration. (**b**) Sevoflurane administration patterns by procedure type and monitoring group.

Anesthesia duration varied mainly by type of surgical procedure, with minimal difference between monitoring strategies (Scheme 1a). Sevoflurane exposure showed a clear left-shift toward reduced use in EG cases (Scheme 1b), consistent with EEG-guided titration.

### 3.2. Hemodynamic Outcomes

Entropy-guided anesthesia was associated with more stable intraoperative hemodynamics. Patients monitored with entropy experienced fewer hypotensive episodes, with the absence of EEG monitoring substantially increasing the likelihood of repeated hypotension during anesthesia. Orthopedic procedures were associated with a lower risk of hypotension than abdominal surgeries.

Mean intraoperative norepinephrine requirements were lower in the EG group, in line with reduced vasopressor dependence. Full statistical outputs are summarized in Supplementary Material S1.

### 3.3. Postoperative Cognitive Outcomes

Early postoperative cognitive dysfunction (NEECHAM ≤ 24) was common at 24 h in the overall cohort, affecting almost half of patients. The prevalence of cognitive impairment decreased steadily over 72 h, with most patients returning to normal or near-normal cognitive function by postoperative day three (Table 4).

### 3.4. Burst Suppression and Additional Predictors of Postoperative Cognitive Decline

Within the entropy-monitored cohort, patients who developed early postoperative cognitive dysfunction (NEECHAM ≤ 24) exhibited significantly greater intraoperative EEG burst suppression. Table 5 summarizes the burst-suppression characteristics in this cohort. Specifically, their mean BSR values range from approximately 27% to 30%, compared with 11% to 14% in cognitively unaffected patients, and mean burst-suppression duration was approximately 9–10 min versus about 5 min in unaffected individuals (*p* < 0.001 for both), as shown in Tables S12 and S13 (Supplementary Material S2).

Intraoperative medication profiles also showed statistical associations with cognitive outcomes. Cases with lower NEECHAM scores at 24 h tended to have received higher doses of fentanyl (*p* < 0.05) and sevoflurane (*p* < 0.01), whereas norepinephrine administration did not demonstrate a significant relationship, as detailed in Tables S8 and S9 (Supplementary Material S2). Importantly, these represent observed correlations and cannot be interpreted as evidence of causation.

**Figure 1:**
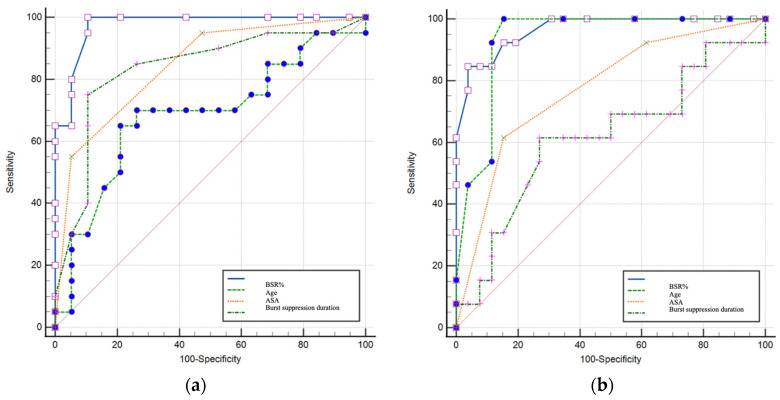
(**a**) ROC performance of EEG-derived and clinical variables for identifying cognitive impairment at 24 h; (**b**) ROC performance of EEG-derived and clinical variables for identifying cognitive impairment at 48 h.

ROC analysis supported the predictive value of burst-suppression metrics. A BSR exceeding 15% and suppression duration over 6 min were associated with cognitive dysfunction at 24 h, while BSR values above 25% were associated with impairment at 48 h. ROC analyses demonstrated strong discriminative performance of burst-suppression metrics for early cognitive impairment; nevertheless, these thresholds should be considered hypothesis-generating and require external validation. Findings are summarized in Table 6 and Table 7 and illustrated in Figure 1a (24 h prediction) and Figure 1b (48 h prediction), with detailed numerical models provided in Tables S10 and S11 (Supplementary Material S2).

In multivariable analysis restricted to the entropy-monitored cohort, burst-suppression burden showed the strongest statistical association with early postoperative cognitive impairment; however, effect size estimates should be interpreted cautiously given the exploratory nature of the model and the limited sample size (Tables S14 and S15 in Supplementary Material S2).

Collectively, these findings indicate that the severity and cumulative duration of intraoperative burst suppression were the strongest markers associated with early postoperative cognitive vulnerability in this population. Among entropy-monitored patients, the absence of documented burst suppression was associated with preserved early postoperative cognitive performance.

## 4. Discussion

### 4.1. Interpretation of Main Findings

The role of intraoperative processed EEG-based anesthetic depth monitoring in emergency surgical settings remains insufficiently characterized, despite well-documented benefits in elective procedures. Emergency surgery patients carry a higher physiological stress load and frequently present with acute pathologies and multiple comorbidities, potentially increasing their vulnerability to postoperative neurocognitive complications. Processed EEG-based anesthetic depth monitoring is not yet standard practice in emergency surgery but is applied selectively based on clinician preference and equipment availability. This real-world variability reflects current clinical practice but also introduces inherent challenges in assessing its impact.

Processed EEG monitoring systems, including Entropy (State Entropy [SE] and Response Entropy [RE]) and the Bispectral Index (BIS), share the common objective of guiding anesthetic depth by analyzing cortical electrical activity. Both Entropy and BIS values decrease with increasing anesthetic depth and are correlated with clinical markers of unconsciousness in surgical anesthesia, including loss of eyelash reflex and loss of consciousness [20]. Entropy monitoring provides complementary information, with SE reflecting primarily cortical EEG activity and RE incorporating additional high-frequency (EMG) activity, which can help detect changes in responsiveness at lighter planes of anesthesia [21]. Comparative studies indicate that while BIS and SE/RE generally agree during induction and maintenance of anesthesia, specific EEG patterns and artifacts—including burst suppression and slow delta activity—can lead to quantitative discrepancies between the two indices due to differences in algorithm design and signal processing [22]. While both BIS and Entropy provide processed EEG indices to guide anesthetic depth, Entropy uniquely provides separate SE and RE metrics that may enhance detection of lighter anesthesia planes via EMG activity. However, differences in algorithm design can lead to variability in burst suppression quantification, which clinicians should consider when interpreting intraoperative EEG data. Both monitoring systems reliably detect EEG signatures associated with deep anesthesia and have been shown to correlate strongly across surgical populations, although thresholds and temporal sensitivity may differ [23]. Awareness of these subtle differences enables clinicians to better interpret burst suppression data and optimize anesthetic titration.

Previous studies have reported variable reductions in anesthesia duration with intraoperative EEG monitoring, ranging from 0.6 to 12 min [24,25,26]. Such variability likely reflects differences in emergence criteria (e.g., eye-opening, extubation, or full orientation). In our retrospective cohort, entropy-guided anesthesia was associated with a mean reduction of approximately 3 min until extubation—a difference that did not reach statistical significance, aligning with observations from Vance et al. [27], who also reported modest or negligible effects on emergence time. These findings suggest that anesthesia duration may be more strongly influenced by surgical procedural factors than by depth-monitoring strategy.

Our analysis revealed a significant association between entropy monitoring and reduced intraoperative consumption of volatile anesthetics and opioids. Sevoflurane usage decreased by approximately 2.3 mL/h, comparable to reductions previously described in elective surgery populations [26,28]. Similarly, fentanyl administration was reduced by approximately 68 µg, consistent with findings by Recart et al. [29]. While rocuronium requirements showed minor differences depending on procedure type, the overall trend indicates reduced anesthetic and neuromuscular depressant exposure associated with EEG-guided titration.

Interestingly, only abdominal emergency procedures required higher crystalloid volumes overall, whereas entropy-monitored cases received significantly less intravenous fluid (~300 mL less). This may relate to decreased volatile anesthetic exposure in the entropy group, potentially reducing the need for compensatory fluid administration aimed at counteracting anesthetic-induced vasodilation [30]. A similar trend was noted for vasopressor use, with lower norepinephrine requirements in the EEG-monitored group, supporting prior evidence linking depth-adjusted anesthesia with preserved vascular tone [31].

### 4.2. Hemodynamic Outcomes

Previous literature has described improved intraoperative hemodynamic stability in patients monitored with depth-of-anesthesia EEG, including reductions in hypotension and bradycardia [32,33]. Definitions of intraoperative hypotension vary, but modern guidelines emphasize maintaining MAP above 60–70 mmHg or avoiding sustained (>10 min) decreases of >20% from baseline [34,35].

In our retrospective cohort, entropy-monitored patients were approximately five times less likely to experience intraoperative hypotension and required lower cumulative norepinephrine doses. These associations suggest that EEG-guided anesthetic titration may contribute to improved hemodynamic stability, possibly by preventing excessively deep anesthetic states.

### 4.3. Postoperative Cognitive Outcomes

Postoperative cognitive status was evaluated using the NEECHAM Confusion Scale, which captures both hyperactive and hypoactive cognitive disturbance and identifies patients at risk of developing delirium [36]. Although assessments were performed once daily—potentially underestimating fluctuating or transient episodes—this schedule permitted systematic monitoring in a high-acuity emergency environment.

Our findings align with an increasing number of studies reporting reduced incidence of postoperative delirium and cognitive impairment in patients receiving EEG-guided anesthetic management [37,38,39,40,41], although guideline recommendations remain moderate (Grade B) due to heterogeneity in study methodologies [2,41]. The inherently high-risk demographic profile of our cohort—median age > 60 years for abdominal cases and >50 years for orthopedic cases, with >80% of the sample presenting ASA ≥ III—likely contributed to the overall incidence of cognitive dysfunction observed [42,43].

In addition to depth titration, processed EEG monitoring enabled quantification of burst suppression ratio (BSR), a marker of deep cortical inactivation that has been linked to adverse neurological outcomes in perioperative populations [8,9,44]. Notably, we observed no significant correlation between intraoperative hypotension and BSR metrics, supporting the interpretation that burst suppression characteristics may reflect underlying neurocognitive vulnerability rather than a direct toxic effect of anesthetic dosing, a distinction that is particularly relevant in high-risk emergency surgery populations.

Importantly, both the frequency and cumulative duration of burst suppression were associated with early postoperative cognitive decline—findings supported by ROC-derived thresholds indicating strong predictive performance. Patients exhibiting burst suppression demonstrated significantly higher risk of cognitive impairment within the first 24–48 postoperative hours, whereas patients without burst suppression showed no measurable decline on NEECHAM evaluation. While our study did not directly quantify burst suppression in relation to specific anesthetic concentrations such as MAC, emerging evidence suggests that burst suppression may reflect underlying cerebral vulnerability rather than simply excessive anesthetic dosing. Reduced frontal alpha power during anesthesia has been linked to an increased propensity for burst suppression, potentially characterizing a “vulnerable brain” phenotype associated with neurocognitive risk [45]. Moreover, systematic reviews indicate that intraoperative burst suppression is associated with an elevated risk of postoperative delirium across surgical cohorts [46]. In addition, EEG dynamics showing decreased alpha and beta power can precede and predict burst suppression in high-risk populations such as cardiac surgery patients, suggesting sensitivity to anesthetic and physiological stress beyond dose alone [47]. Case reports and related evidence further support that certain EEG patterns linked to vulnerability may manifest at lower anesthetic concentrations, consistent with the concept that individual brain sensitivity contributes to suppression occurrence [47,48].

### 4.4. Clinical Implications

To our knowledge, few studies have focused on emergency surgery populations, making this retrospective investigation a valuable contribution. Our results highlight the feasibility and potential benefits of intraoperative EEG-based anesthetic depth monitoring in high-risk surgical patients. Entropy-guided anesthesia was associated with reduced anesthetic and opioid exposure, along with modest changes in fluid and vasopressor management, supporting its practical utility in routine emergency surgical care.

### 4.5. Cost Effectiveness Considerations

While no formal economic analysis was conducted in this study, the observed reductions in anesthetic and opioid consumption, along with improved hemodynamic stability, suggest potential cost benefits. Nevertheless, upfront equipment costs and availability remain important considerations, especially in emergency surgery contexts. Future prospective studies should explicitly evaluate the cost-effectiveness of entropy monitoring to better inform clinical practice.

### 4.6. Study Limitations

This retrospective, observational, single-center cohort study provides valuable insight into the association between entropy-guided anesthesia and early postoperative cognitive outcomes in emergency surgery, yet several limitations warrant emphasis. The observational design precludes causal inference, and unmeasured confounding by indication cannot be excluded given the non-randomized application of entropy monitoring based on clinician preference. The single-institution setting may limit generalizability across broader surgical populations or healthcare environments.

Follow-up was restricted to 72 h postoperatively, capturing short-term cognitive effects but not longer-term neurocognitive trajectories. The heterogeneity of surgical procedures (abdominal vs. orthopedic) may have introduced variability in stress physiology and anesthetic management. Furthermore, detailed data on intraoperative ventilation strategies, including mode of ventilation and use of lung-protective measures such as low tidal volumes and PEEP, were not consistently available in the retrospective records. Additionally, although major neurological, cardiovascular, and metabolic complications were systematically excluded, the retrospective nature of the study limited comprehensive capture of all perioperative adverse events. Consequently, other clinical complications potentially influencing postoperative cognitive outcomes may not have been fully accounted for, representing an inherent limitation in observational emergency surgery research. These factors may influence cerebral perfusion and postoperative recovery and thus represent additional limitations of our study.

Formal preoperative cognitive screening was not available, limiting the ability to establish baseline neurocognitive status. Although residual confounding cannot be excluded, logistic regression analyses adjusting for key anesthetic and clinical variables (Supplementary Tables S8 and S9) suggest that the observed associations between intraoperative management and postoperative cognitive impairment merit further investigation.

Because entropy monitoring was applied according to real-world clinician preference rather than protocolized assignment, residual confounding by indication cannot be excluded. Due to the retrospective design, complete blinding of treating clinicians was impossible, and documentation-based data extraction may be subject to recording bias. Cognitive evaluation relied on once-daily NEECHAM scoring, which may underestimate fluctuating delirium characteristics. Finally, burst-suppression values were obtained from processed EEG via the GE Entropy™ system without review of raw EEG tracings, which may limit physiological specificity.

## 5. Conclusions

In this retrospective cohort study, intraoperative processed EEG-guided anesthesia was associated with reduced exposure to volatile anesthetics, opioids, and neuromuscular blockers, as well as decreased intravenous fluid and vasopressor requirements. These findings suggest that real-time cortical activity monitoring can support more individualized and precise anesthetic management in emergency surgical patients.

Entropy monitoring correlated with fewer hypotensive episodes and more stable arterial pressures, factors critical to maintaining end-organ perfusion and optimizing postoperative recovery. Furthermore, intraoperative burst suppression was strongly linked to early postoperative cognitive impairment, indicating its potential as a clinically accessible marker of neurocognitive vulnerability.

This study provides novel observational evidence supporting the feasibility and clinical value of processed EEG monitoring in emergency surgery. Further prospective, multicenter research is warranted to confirm these findings and to determine how burst suppression metrics might guide personalized anesthetic dosing strategies to improve neurological outcomes in high-risk surgical populations.

## Figures and Tables

**Table 1:** Baseline patient characteristics by monitoring category (EG vs. SG).

Variable	EG (n = 87)	SG (n = 81)	*p*-Value
Age (years), mean (SD)			
General surgery	62.6 (16.3)	58.6 (18.0)	0.15
Orthopedic surgery	51.6 (21.1)	55.8 (18.8)	0.28
Body mass index (kg/m2), mean (SD)			
General surgery	27.6 (4.1)	24.5 (4.2)	0.09
Orthopedic surgery	26.6 (3.7)	26.3 (3.8)	0.78
Male sex, n (%)			
General surgery	60%	54%	0.45
Orthopedic surgery	51%	51%	1.00
ASA physical status, mean (SD)			
General surgery	2.8 (0.76)	2.62 (0.78)	0.20
Orthopedic surgery	2.7 (0.75)	2.7 (0.65)	1.00
Any comorbidity, n (%)			
General surgery	85%	79%	0.32
Orthopedic surgery	85%	83%	0.78
Cardiovascular comorbidity, n (%)			
General surgery/Orthopedic surgery	67%/53%	55%/53%	0.11/1.00
Metabolic comorbidity, n (%)			
General surgery/Orthopedic surgery	45%/25%	26%/20%	0.03/0.54
Renal comorbidity, n (%)			
General surgery/Orthopedic surgery	6%/0%	4%/0%	0.58/—
Pulmonary comorbidity, n (%)			
General surgery/Orthopedic surgery	3%/4%	9%/7%	0.12/0.68
Trauma indication, n (%)			
General surgery/Orthopedic surgery	9%/40%	21%/30%	0.08/0.31
Neurologic comorbidity, n (%)			
General surgery/Orthopedic surgery	21%/15%	15%/13%	0.35/0.71
Number of comorbidities, mean (SD)			
General surgery	1.89 (1.57)	1.69 (1.56)	0.08
Orthopedic surgery	1.57 (—)	1.56 (—)	0.84

**Table 2:** Intraoperative anesthetic exposure and procedure duration.

Variable	Surgery Type	EG (n = 87) Mean (SD)	SG (n = 81) Mean (SD)	*p*-Value
Anesthesia duration (min)	General surgery	157.5 (29.6)	155.0 (30.7)	0.45
	Orthopedic surgery	119.1 (24.3)	124.3 (25.3)	0.28
Fentanyl (µg)	General surgery	458.6 (87.0)	531.0 (87.4)	<0.001
	Orthopedic surgery	373.6 (56.0)	441.7 (73.2)	<0.001
Sevoflurane (mL/h)	General surgery	8.52 (1.03)	10.99 (1.34)	<0.001
	Orthopedic surgery	8.41 (1.26)	10.48 (1.46)	<0.001
Rocuronium (mg)	General surgery	85.3 (17.3)	95.6 (17.3)	0.02
	Orthopedic surgery	52.5 (10.9)	52.0 (10.6)	0.78
Crystalloid (mL)	General surgery	2928.5 (875.6)	3330.0 (718.6)	0.03
	Orthopedic surgery	2471.7 (901.0)	2683.3 (1029.5)	0.25
Colloid (mL)	General surgery	657.1 (338.1)	740.0 (338.7)	0.12
	Orthopedic surgery	518.9 (391.8)	583.3 (416.9)	0.30
Blood products (units)	General surgery	0.46 (0.51)	0.72 (0.54)	0.04
	Orthopedic surgery	0.58 (0.66)	0.73 (0.58)	0.28
Perioperative morphine (>0.1 mg/kg), n (%)	General surgery	63%	78%	0.03
	Orthopedic surgery	0%	0%	–
Noradrenaline (µg/kg/min)	General surgery	0.14 (0.25)	0.25 (0.30)	0.04
	Orthopedic surgery	0.06 (0.16)	0.15 (0.29)	0.08
Regional analgesia, n (%)	General surgery	9%	10%	0.81
	Orthopedic surgery	81%	77%	0.62

**Table 3:** Perioperative fluid administration and vasopressor use.

Variable	Type of Surgery	EG (n = 87) Mean (SD)	SG (n = 81) Mean (SD)	*p*-Value
**Crystalloid (mL)**	General surgery	2928.5 (875.6)	3330.0 (718.6)	0.03
	Orthopedic surgery	2471.7 (900.9)	2683.3 (1029.5)	0.28
**Colloid (mL)**	General surgery	657.1 (338.1)	740.0 (338.7)	0.12
	Orthopedic surgery	518.9 (391.8)	583.3 (416.9)	0.38
**Blood products (units)**	General surgery	0.46 (0.51)	0.72 (0.54)	0.05
	Orthopedic surgery	0.58 (0.66)	0.73 (0.58)	0.42
**Noradrenaline (µg/kg/min** **)**	General surgery	0.14 (0.25)	0.25 (0.30)	0.04
	Orthopedic surgery	0.06 (0.16)	0.15 (0.29)	0.22

**Table 4:** Postoperative NEECHAM cognitive assessment across time points.

Time Point	Min–Max	Mean (SD)	Md (Q1–Q3)	Skewness (sk)	Kurtosis (K)	≤24 (%)
24 h	17–30	24.51 (2.78)	25.0 (23–27)	−0.61	−0.19	43.2
48 h	18–30	25.56 (2.86)	26.0 (24–28)	−0.49	−0.21	32.5
72 h	21–30	27.90 (2.10)	28.0 (27–30)	−0.95	0.30	8.3

**Table 5:** Burst suppression metrics in the entropy-monitored cohort (n = 87).

Parameter	Min–Max	Mean (SD)	Median (Q1–Q3)	Skewness (sk)	Kurtosis (K)
BSR (%)	4–41	19.36 (10.02)	18.0 (11–28)	0.41	−0.95
BS Duration (min)	2–15	6.85 (3.53)	6.0 (4–10)	0.63	−0.52

**Table 6:** ROC classification performance of BSR% for identifying early cognitive impairment.

Time Point	AUC [95% CI]	SE	*p*-Value	Youden Index (J)	Cutoff Criterion	Sensitivity (%)	Specificity (%)
24 h	0.975 [0.806–0.999]	0.02	<0.001	0.895	>15%	100.0	89.47
48 h	0.964 [0.850–0.998]	0.03	<0.001	0.808	>25%	84.62	96.15
72 h	-	—	—	—	—	—	—

**Table 7:** ROC classification performance of burst-suppression duration for identifying cognitive impairment.

Time Point	AUC [95% CI]	SE	*p*-Value	Youden Index (J)	Cutoff Criterion	Sensitivity (%)	Specificity (%)
24 h	0.834 [0.681–0.934]	0.07	<0.001	0.645	>6 min	75.0	89.47
48 h	0.933 [0.805–0.988]	0.04	<0.001	0.846	>6 min	100.0	84.62
72 h	-	—	—	—	—	—	—

## Data Availability

The anonymized datasets generated and analyzed during the current study are available within the article and Supplementary Materials. Additional de-identified data may be provided upon reasonable request to the corresponding author. Raw patient-identifiable data cannot be released due to ethical and legal restrictions.

## Supplementary Materials

The following supporting information can be downloaded at: https://www.mdpi.com/article/10.3390/jcm15030968/s1, Supplementary Material S1: Table S1: Predictive model coefficients for sevoflurane dosage. Table S2: Predictive model coefficients for fentanyl use. Table S3: Predictive model coefficients for crystalloid volume. Table S4: Predictive model coefficients for initial hypotension episodes. Table S5: Estimated parameters for intraoperative hypotension episodes. Table S6: Descriptive statistics for intraoperative norepinephrine dose. Table S7: Predictive model coefficients for norepinephrine requirement. Supplementary Material S2: Table S8: Regression model of postoperative cognitive impairment at 24 h. Table S9: Regression model of postoperative cognitive impairment at 48 h. Table S10: ROC analysis at 24 h for BSR(%), BS duration, ASA score, and age. Table S11: ROC analysis at 48 h for BSR(%), BS duration, ASA score, and age. Table S12: BSR% vs. NEECHAM ≤ 24 at 24 and 48 h. Table S13: Burst-suppression duration vs. NEECHAM ≤ 24 at 24 and 48 h. Table S14: Logistic regression predicting NEECHAM ≤ 24 at 24 h. Table S15: Logistic regression predicting NEECHAM ≤ 24 at 48 h.

## References

[1] Radtke F.M., Franck M., Lendner J., Krüger S., Wernecke K.D., Spies C.D. (2013). Monitoring depth of anaesthesia in a randomized trial decreases the rate of postoperative delirium but not postoperative cognitive dysfunction. Br. J. Anaesth..

[2] Aldecoa C., Bettelli G., Bilotta F., Sanders R.D., Aceto P., Audisio R., Cherubini A., Cunningham C., Dabrowski W., Forookhi A. (2024). Update of the European Society of Anaesthesiology and Intensive Care Medicine evidence-based and consensus-based guideline on postoperative delirium in adult patients. Eur. J. Anaesthesiol..

[3] Revue Médicale de Liège-Monitorage de la Profondeur de l’Anesthésie: Pourquoi, Comment et à Quel Prix?. https://rmlg.uliege.be/article/1656?lang=en.

[4] Wang F., Wang Y., Wu H., Lei L., Xu S., Shen X., Guo X., Shen R., Xia X., Liu Y. (2014). Postoperative Cognitive Dysfunction: Current Developments in Mechanism and Prevention. Med. Sci. Monit..

[5] Janssen T.L., Alberts A.R., Hooft L., Mattace-Raso F.U.S., Mosk C.A., Van Der Laan L. (2019). Prevention of postoperative delirium in elderly patients planned for elective surgery: Systematic review and meta-analysis. Clin. Interv. Aging.

[6] Musizza B., Ribaric S. (2010). Monitoring the Depth of Anaesthesia. Sensors.

[7] Pedemonte J.C., Plummer G.S., Chamadia S., Locascio J.J., Hahm E., Ethridge B., Gitlin J., Ibala R., Mekonnen J., Colon K.M. (2020). Electroencephalogram Burst-suppression during Cardiopulmonary Bypass in Elderly Patients Mediates Postoperative Delirium. Anesthesiology.

[8] Soehle M., Dittmann A., Ellerkmann R.K., Baumgarten G., Putensen C., Guenther U. (2015). Intraoperative burst suppression is associated with postoperative delirium following cardiac surgery: A prospective, observational study. BMC Anesthesiol..

[9] Fritz B.A., Maybrier H.R., Avidan M.S. (2018). Intraoperative electroencephalogram suppression at lower volatile anaesthetic concentrations predicts postoperative delirium occurring in the intensive care unit. Br. J. Anaesth..

[10] Schuller P.J., Pretorius J.P.G., Newbery K.B. (2023). Response of the GE Entropy^TM^ monitor to neuromuscular block in awake volunteers. Br. J. Anaesth..

[11] Sinha P.K.K.T. (2007). Monitoring Devices for Measuring the Depth of Anaesthesia. Indian J. Anaesth..

[12] Steyn-Ross M.L., Steyn-Ross D.A., Sleigh J.W., Wilcocks L.C. (2001). Toward a theory of the general-anesthetic-induced phase transition of the cerebral cortex. I. A thermodynamics analogy. Phys. Rev. E.

[13] Wheeler P., Hoffman W.E., Baughman V.L., Koenig H. (2005). Response entropy increases during painful stimulation. J. Neurosurg. Anesthesiol..

[14] Vakkuri A., Yli-Hankala A., Talja P., Mustola S., Tolvanen-Laakso H., Sampson T., Viertiö-Oja H. (2004). Time-frequency balanced spectral entropy as a measure of anesthetic drug effect in central nervous system during sevoflurane, propofol, and thiopental anesthesia. Acta Anaesthesiol. Scand..

[15] Viertiö-Oja H., Maja V., Särkelä M., Talja P., Tenkanen N., Tolvanen-Laakso H., Paloheimo M., Vakkuri A., Yli-Hankala A., Meriläinen P. (2004). Description of the EntropyTM algorithm as applied in the Datex-Ohmeda S/5TM Entropy Module. Acta Anaesthesiol. Scand..

[16] Neelon V.J., Champagne M.T., Carlson J.R., Funk S.G. (1996). The NEECHAM Confusion Scale: Construction, validation, and clinical testing. Nurs. Res..

[17] Immers H.E.M., Schuurmans M.J., Van De Bijl J.J. (2005). Recognition of delirium in ICU patients: A diagnostic study of the NEECHAM confusion scale in ICU patients. BMC Nurs..

[18] Joshi S. (2007). Current concepts in the management of delirum. Mo Med..

[19] Sawamura S., Morimoto Y. (2017). Diagnosis of POD and POCD. Anesthesia and Neurotoxicity.

[20] Gao J.-D., Zhao Y.-J., Xu C.-S., Zhao J., Huang Y.-G., Wang T.-L., Pei L., Wang J., Yao L.-N., Ding Q. (2012). Evaluation of entropy for monitoring the depth of anesthesia compared with bispectral index: A multicenter clinical trial. Chin. Med. J..

[21] Knudsen K. (2025). Anesthesia Depth Monitoring—BIS, SED Line and Entropy in Anesthesia Monitoring. The Anesthesia Guide. https://anesthguide.com/topic/anesthesia-depth-monitoring-bis/.

[22] Aho A., Kamata K., Jäntti V., Kulkas A., Hagihira S., Huhtala H., Yli-Hankala A. (2015). Comparison of Bispectral Index and Entropy values with electroencephalogram during surgical anaesthesia with sevoflurane. Br. J. Anaesth..

[23] Baulig W., Seifert B., Schmid E.R., Schwarz U. (2010). Comparison of spectral entropy and bispectral index electroencephalography in coronary artery bypass graft surgery. J. Cardiothorac. Vasc. Anesth..

[24] Bresil P., Nielsson M.S., Malver L.P., Kraemer K., Schjørring O., Dethlefsen C., Lambert P.H. (2013). Impact of Bispectral Index for monitoring propofol remifentanil anaesthesia. A randomised clinical trial. Acta Anaesthesiol. Scand..

[25] Oliveira C.R.D., Bernardo W.M., Nunes V.M. (2017). Benefit of general anesthesia monitored by bispectral index compared with monitoring guided only by clinical parameters. Systematic review and meta-analysis. Braz. J. Anesthesiol..

[26] Chan M.T.V., Cheng B.C.P., Lee T.M.C., Gin T. (2013). BIS-guided anesthesia decreases postoperative delirium and cognitive decline. J. Neurosurg. Anesthesiol..

[27] Vance J.L., Shanks A.M., Woodrum D.T. (2014). Intraoperative bispectral index monitoring and time to extubation after cardiac surgery: Secondary analysis of a randomized controlled trial. BMC Anesthesiol..

[28] Cotae A.M., Cobilinschi C., Băetu A.E., Iacob D.M., Grinţescu I.M. (2021). The Impact of Monitoring Depth of Anesthesia and Nociception on Postoperative Cognitive Function in Adult Multiple Trauma Patients. Medicina.

[29] Recart A., Gasanova I., White P.F., Thomas T., Ogunnaike B., Hamza M., Wang A. (2003). The Effect of Cerebral Monitoring on Recovery after General Anesthesia: A Comparison of the Auditory Evoked Potential and Bispectral Index Devices with Standard Clinical Practice. Anesth. Analg..

[30] Juhász M., Molnár L., Fülesdi B., Végh T., Páll D., Molnár C. (2019). Effect of sevoflurane on systemic and cerebral circulation, cerebral autoregulation and CO_2_ reactivity. BMC Anesthesiol..

[31] Nitzschke R., Wilgusch J., Kersten J.F., Trepte C.J., Haas S.A., Reuter D.A., Goepfert M.S. (2014). Bispectral index guided titration of sevoflurane in on-pump cardiac surgery reduces plasma sevoflurane concentration and vasopressor requirements: A prospective, controlled, sequential two-arm clinical study. Eur. J. Anaesthesiol..

[32] Punjasawadwong Y., Phongchiewboon A., Bunchungmongkol N. (2014). Bispectral index for improving anaesthetic delivery and postoperative recovery. Cochrane Database Syst. Rev..

[33] Shalbaf R., Behnam H., Jelveh Moghadam H. (2015). Monitoring depth of anesthesia using combination of EEG measure and hemodynamic variables. Cogn. Neurodynamics.

[34] Brady K.M., Hudson A., Hood R., DeCaria B., Lewis C., Hogue C.W. (2020). Personalizing the Definition of Hypotension to Protect the Brain. Anesthesiology.

[35] Halvorsen S., Mehilli J., Cassese S., Hall T.S., Abdelhamid M., Barbato E., De Hert S., de Laval I., Geisler T., Hinterbuchner L. (2022). 2022 ESC Guidelines on cardiovascular assessment and management of patients undergoing non-cardiac surgery. Eur. Heart J..

[36] Van Rompaey B., Schuurmans M.J., Shortridge-Baggett L.M., Truijen S., Elseviers M., Bossaert L. (2008). A comparison of the CAM-ICU and the NEECHAM confusion scale in intensive care delirium assessment: An observational study in non-intubated patients. Crit. Care.

[37] Chen Y.-C., Hung I.-Y., Hung K.-C., Chang Y.-J., Chu C.-C., Chen J.-Y., Ho C.-H., Yu C.-H. (2023). Incidence change of postoperative delirium after implementation of processed electroencephalography monitoring during surgery: A retrospective evaluation study. BMC Anesthesiol..

[38] Evered L.A., Chan M.T., Han R., Chu M.H., Cheng B.P., Scott D.A., Pryor K.O., Sessler D.I., Veselis R., Frampton C. (2021). Anaesthetic depth and delirium after major surgery: A randomised clinical trial. Br. J. Anaesth..

[39] Pérez-Otal B., Aragón-Benedí C., Pascual-Bellosta A., Ortega-Lucea S., Martínez-Ubieto J., Ramírez-Rodríguez J.M., Quesada-Gimeno N., Muñoz-Rodríguez L.A., Jiménez-Bernadó T., Pérez-Navarro G. (2022). Neuromonitoring depth of anesthesia and its association with postoperative delirium. Sci. Rep..

[40] Miao M., Xu Y., Sun M., Chang E., Cong X., Zhang J. (2020). BIS index monitoring and perioperative neurocognitive disorders in older adults: A systematic review and meta-analysis. Aging Clin. Exp. Res..

[41] Ormseth C.H., LaHue S.C., Oldham M.A., Josephson S.A., Whitaker E., Douglas V.C. (2023). Predisposing and Precipitating Factors Associated with Delirium. JAMA Netw. Open.

[42] Jin Z., Hu J., Ma D. (2020). Postoperative delirium: Perioperative assessment, risk reduction, and management. Br. J. Anaesth..

[43] Fritz B.A., Kalarickal P.L., Maybrier H.R., Muench M.R., Dearth D., Chen Y., Escallier K.E., Ben Abdallah A., Lin N., Avidan M.S. (2016). Intraoperative Electroencephalogram Suppression Predicts Postoperative Delirium. Anesth. Analg..

[44] Shao Y.R., Kahali P., Houle T.T., Deng H., Colvin C., Dickerson B.C., Brown E.N., Purdon P.L. (2020). Low Frontal Alpha Power Is Associated with the Propensity for Burst Suppression: An Electroencephalogram Phenotype for a ”Vulnerable Brain”. Anesth. Analg..

[45] Park S.K., Han D.W., Chang C.H., Jung H., Kang H., Song Y. (2025). Association between Intraoperative Electroencephalogram Burst Suppression and Postoperative Delirium: A Systematic Review and Meta-analysis. Anesthesiology.

[46] Plummer G.S., Ibala R., Hahm E., An J., Gitlin J., Deng H., Shelton K.T., Solt K., Qu J.Z., Akeju O. (2019). Electroencephalogram dynamics during general anesthesia predict the later incidence and duration of burst-suppression during cardio-pulmonary bypass. Clin. Neurophysiol..

[47] Wakabayashi R. (2023). Anesthetic management of a patient with an electroencephalogram phenotype for a “vulnerable brain”: A case report. JA Clin. Rep..

[48] Schwerin S., Schneider G., Kreuzer M., Kratzer S. (2024). Impact of Age on the Occurrence of Processed Electroencephalographic Burst Suppression. Anesth. Analg..

