# Supplementary material for: Association Between Entropy Monitoring, Burst Suppression and Early Postoperative Cognitive Dysfunction in Emergency Surgery: A Retrospective Cohort Study
Source: J Clin Med. 2026 Jan 25;15(3):968. doi: 10.3390/jcm15030968 (PMC12898030; doi:10.3390/jcm15030968)
Supplement: Supplementary file 1 [file jcm-15-00968-s001.zip › jcm-4085726-supplementary.zip!/jcm-4085726-supplementary.pdf]

Supplementary Material S1

Table S1 *Predictive model coefficients for sevoflurane dosage based on entropy monitoring and surgical intervention type.*

Model	Unstandardized Coefficients B	Std. Error	Standardized Coefficients Beta	T	Sig.	95.0% Confidence Interval for B Lower Bound	Upper Bound
(Constant)	10.59	.19		55.52	.000	10.21	10.97
Entropy	-2.31	.20	-.661	-11.41	.000	-2.71	-1.91
Type of intervention	.34	.20	.096	1.65	.100	-.07	.73

a. Dependent Variable: Sevoflurane(ml/h)

Table S2 *Predictive model coefficients for fentanyl usage in relation to entropy monitoring and surgical intervention type.*

Model	Unstandardized Coefficients B	Std. Error	Standardized Coefficients Beta	T	Sig.	95.0% Confidence Interval for B Lower Bound	Upper Bound
(Constant)	441.63	11.08		39.84	.000	419.74	463.51
Entropy	-68.02	11.79	-.358	-5.77	.000	-91.29	-44.75
Type of intervention	84.93	11.77	.448	7.22	.000	61.69	108.17

a. Dependent Variable: Fentanyl (µg)

Table S3 *Predictive model coefficients for crystalloid fluid volume according to entropy monitoring and surgical intervention type.*

Model	Unstandardized Coefficients B	Std. Error	Standardized Coefficients Beta	t	Sig.	95.0% Confidence Interval for B Lower Bound	Upper Bound
(Constant)	2746.11	129.83		21.15	.000	2489.76	3002.46
Entropy	-309.95	137.89	-.167	-2.25	.026	-582.20	-37.70
Type of intervention	546.22	137.74	.294	3.97	.000	274.26	818.18

Dependent Variable: Crystalloid (ml)

Table S4 *Predictive model coefficients for initial intraoperative hypotension episodes in relation to entropy monitoring and surgical intervention type.*

	B	S.E.	Wald	df	Sig.	Exp(B)	95% C.I.for EXP(B)	
							Lower	Upper
Entropy (1)	-.21	.32	.43	1	.510	.81	.43	1.52
Type of intervention (1)	.86	.32	7.02	1	.008	2.36	1.25	4.45
Constant	-.49	.31	2.53	1	.112	.61		

a. Variable(s) entered on step 1: Entropy, Type of intervention.

Table S5 *Estimated parameters for the predictive model of intraoperative hypotension episodes based on entropy monitoring and surgical intervention type.*

		B	Std. Error	Wald	df	Sig.	Exp(B)	95% Confidence Interval Exp(B)	
								Lower Bound	Upper Bound
Location	[Entropy_p=0]	1.70	.31	29.20	1	.000	5.42	2.91	10.10
	[Entropy_p=1]	0[a]	.	.	0	.		.	.
	[Orthopedic=0]	-.88	.30	8.88	1	.003	.40	.22	.71
	[General Surgery=1]	0[a]	.	.	0	.		.	.

Table S6 *Descriptive statistics of intraoperative norepinephrine dosage.*

	min-max	m±sd	md (Q1 – Q3)	sk	K
Noradrenaline	.01 – 1.00	.35±.30	.30 (.07 – .60)	.51	-1.10

Table S7 *Predictive model coefficients for intraoperative norepinephrine dosage associated with entropy monitoring.*

Model	Unstandardized Coefficients		Standardized Coefficients	t	Sig.
	B	Std. Error	Beta		
(Constant)	.41	.05		9.08	.000
Entropy	-.14	.07	-.24	-2.05	.044

Supplementary Material S2

Table S8 *Logistic regression model of postoperative cognitive impairment at 24 hours: impact of fentanyl, sevoflurane, and norepinephrine.*

Factor	Hosmer & Lemeshow χ^2 (df)	R^2 (Cox & Snell – Nagelkerke)	B [SE]	Wald (df=1)	p-value	Exp(B)	95% CI
f1 (Norepinephrine)	17.36 (8)	0.067	-0.04 [0.18]	0.05	0.82	0.96	0.69 – 1.38
f2 (Fentanyl)			5.51	0.019	1.59	1.08 – 2.35	0.47 [0.20]
f3 (Sevoflurane)			7.26	0.007	1.67	1.15 – 2.43	0.51 [0.19]

Note: *Hosmer & Lemeshow χ^2 test assesses model goodness of fit; R^2 indicates model fit (Cox & Snell – Nagelkerke); B is the regression coefficient with standard error [SE]; Wald is the Wald statistic; Exp(B) is the odds ratio with 95% confidence interval (CI).*

Table S9 *Logistic regression model of postoperative cognitive impairment at 48 hours: impact of fentanyl, sevoflurane, and norepinephrine.*

Factor	Hosmer & Lemeshow χ^2 (df)	p (HL)	R^2 (Cox & Snell – Nagelkerke)	B [SE]	Wald (df=1)	p-value	Exp(B)	95% CI
f1 (Norepinephrine)	6.84 (8)	0.554	0.076 – 0.106	-0.11 [0.19]	0.31	0.580	0.90	0.62 – 1.31
f2 (Fentanyl)				0.45 [0.20]	4.93	0.026	1.56	1.05 – 2.32
f3 (Sevoflurane)				0.32 [0.19]	2.83	0.092	1.38	0.95 – 2.01

Note: *Hosmer & Lemeshow χ^2 test assesses goodness of fit; R^2 indicates model fit (Cox & Snell – Nagelkerke); B is the regression coefficient with standard error [SE]; Wald is the Wald statistic; Exp(B) is the odds ratio with 95% confidence interval (CI).*

Table S10 *Comparative ROC analysis at 24 hours for burst suppression ratio (BSR %), burst suppression duration, ASA physical status, and patient age.*

Factor	AUC	SE	Significance vs. BSR (%)	vs. BS duration	vs. ASA score	vs. Age
BSR(%)	0.975	0.02	–	–	–	–
BS duration	0.834	0.07	0.040	–	–	–
ASA score	0.843	0.06	0.030	0.921	–	–
Age	0.684	0.09	0.001	0.185	0.103	–

Table S11 *Comparative ROC analysis at 48 hours for burst suppression ratio (BSR %), burst suppression duration, ASA physical status, and patient age.*

Factor	AUC	SE	Significance vs. BSR (%)	vs. BS duration	vs. ASA score	vs. Age
BSR(%)	0.964	0.03	–	–	–	–
BS duration	0.933	0.04	0.489	–	–	–
ASA score	0.772	0.08	0.006	0.089	–	–
Age	0.617	0.10	0.001	0.006	0.145	–

Table S12 *Comparative analysis of burst suppression ratio (BSR %) and NEECHAM cognitive scores ≤24 at 24 and 48 hours postoperatively.*

Timepoint	Neecham Score	Range (min–max)	Mean (SD)	t (df=37)	p-value
24 h	>24	4-24	11.26 (4.63)	-8.06	<0.001
	≤ 24	16-41	27.05 (7.25)		
48 h	>24	4-28	13.85 (6.21)	-7.80	<0.001
	≤ 24	18-41	30.38 (6.31)		

Table S13 *Comparative analysis of burst suppression duration and NEECHAM cognitive scores ≤24 at 24 and 48 hours postoperatively.*

Timepoint	Neecham Score	Range (min–max)	Mean (SD)	t (df=37)	p-value
24 h	>24	2-12	4.89 (2.51)	-3.97	<0.001
	≤ 24	2-15	8.70 (3.39)		
48 h	>24	2-12	5.12(2.49)	-6.01	<0.001
	≤ 24	7-15	10.31 (2.66)		

Table S14 *Binary logistic regression predicting presence of NEECHAM cognitive impairment (score ≤24) at 24 hours postoperatively.*

Factor	Hosmer & Lemeshow			R^2 (Cox&Snell – Nagelkerke)	B [SE]	Wald (df=1)	p	Exp(B)	CI 95%
	χ^2	df	P						
f1(Age)	1.67	8	0.990	0.542 – 0.807	0.88 [0.53]	2.69	0.101	2.40	0.84 – 6.83
f2 (Number of BSR events)					5.60 [2.65]	4.45	0.035	269.48	1.5 – 488
f3 (Duration of BSR events)					0.14 [0.16]	0.77	0.381	1.15	0.84 – 1.58
f4 (Intra-anesthetic hypotension)					0.46 [0.51]	0.80	.371	1.58	0.58 – 4.28
f5 (Peri-operative metabolic acidosis)					2.59 [1.37]	3.58	0.058	13.37	0.91 – 195.9

Note: *χ^2 – Chi-square test value; R^2 – model fit coefficient; B (SE) – regression coefficient (Standard Error); Exp(B) – odds ratio.*

Table S15 *Binary logistic regression predicting presence of NEECHAM cognitive impairment (score ≤24) at 48 hours postoperatively.*

Factor	Hosmer & Lemeshow			R^2 (Cox&Snell – Nagelkerke)	B±SE	Wald (df=1)	p	Exp(B)	CI 95%
	χ^2	df	P						
f1 (Age)	1.00	8	0.998	0.489 -0 .810	0.11 [0.59]	0.03	0.857	1.11	0.35– 3.58
f2 (Number of BSR events)					1.59 [2.20]	0.52	0.470	4.89	0.07 – 0.365
f3 (Duration of BSR events)					0.50 [0.24]	4.52	0.034	1.65	1.04 – 2.63
f4 (Intra-anesthetic hypotension)					0.78 [0.64]	1.49	0.222	2.17	0.63 – 7.54

f5 (Peri-operative metabolic acidosis)					0.67 [1.28]	0.28	0.600	1.96	0.16 – 23.99

Note: *χ^2 – Chi-square test value; R^2 – model fit coefficient; B (SE) – regression coefficient (Standard Error); Exp(B) – odds ratio.*
